# CD19嵌合抗原受体T细胞靶向B细胞肿瘤细胞株过程中糖皮质激素对其扩增影响的体外研究

**DOI:** 10.3760/cma.j.issn.0253-2727.2021.09.006

**Published:** 2021-09

**Authors:** 昊彬 邓, 美静 刘, 嫣雨 江, 婷 袁, 蕊 张, 琦 邓

**Affiliations:** 天津医科大学一中心临床学院，天津市第一中心医院血液科，南开大学医学院 300192 The First Central Clinical College of Tianjin Medical University, Department of Hematology, Tianjin First Central Hospital, School of Medicine, Nankai University, Tianjin 300192, China

**Keywords:** 糖皮质激素, 嵌合抗原受体T细胞, 增殖, Glucocorticoid, Chimeric antigen receptor engineered T cell, Proliferation

## Abstract

**目的:**

评估糖皮质激素对CD19嵌合抗原受体T细胞（CAR-T细胞）靶向杀瘤过程中增殖的影响。

**方法:**

健康志愿者的外周血单个核细胞（PBMC）作为T细胞来源，经CD3磁珠分选及CD19 CAR慢病毒转染制备CD19 CAR-T细胞；采用流式细胞术（FCM）测定CD19 CAR-T细胞转染率及其在培养体系中所占比例；采用CFDA SE细胞增殖示踪荧光探针试剂标记CD19 CAR-T细胞后测定其荧光强度；LDH细胞毒性检测方法检测不同浓度的糖皮质激素对B细胞肿瘤细胞株杀伤活性的影响。

**结果:**

①CD19 CAR-T细胞CD19 CAR转染率为（51.34±5.28）％。②24 h后不同剂量甲泼尼龙对Nalm6、Pfeiffer及U2932细胞的杀伤活性高于地塞米松，48 h后低浓度（4 mg/ml）甲泼尼龙对Nalm6、Pfeiffer、U2932细胞杀伤活性高于低浓度（0.75 mg/ml）地塞米松，而高浓度（12 mg/ml）甲泼尼龙杀伤活性低于高浓度（2.25 mg/ml）地塞米松。但各个时间不同剂量甲泼尼龙组对EHEB细胞的杀伤活性均低于对应时间、剂量的地塞米松。③CD19 CAR-T细胞在不同浓度糖皮质激素作用下的比例及平均荧光强度比较，地塞米松对CD-19 CAR-T细胞的增殖抑制作用强于甲泼尼龙，两种糖皮质激素的高浓度组较低浓度组对CD19 CAR-T细胞的增殖抑制作用更明显。④CD19 CAR-T细胞与不同肿瘤细胞共培养体系中，其比例及平均荧光强度比较，地塞米松对CD19 CAR-T细胞的增殖抑制作用强于甲泼尼龙，两种糖皮质激素的高浓度组较低浓度组对CD19 CAR-T细胞的增殖抑制更明显。

**结论:**

CD19 CAR-T细胞靶向不同肿瘤细胞株过程中地塞米松对CD19 CAR-T细胞的扩增和增殖抑制作用大于甲泼尼龙，高剂量组该抑制作用更加明显。

## Abstract (en)

**Objective:**

To evaluate the effects of glucocorticoids (dexamethasone and methylprednisolone) on the proliferation of CD19 Chimeric antigen receptor (CAR) modified T cells in vitro.

**Methods:**

Peripheral blood mononuclear cells from healthy volunteers were collected as T cells. CD19 CAR-T cells were prepared by CD3 magnetic beads sorting and CD19 CAR lentivirus transfection. The transfection rates and the proportion of CD19 CAR-T cells in the culture system were analyzed using a flow cytometer. The mean fluorescence intensity (MFI) of CD19 CAR-T cells was measured after staining with Carboxyfluorescein diacetate succinimidyl ester cell proliferation tracer fluorescent probe, Lactate dehydrogenase (LDH) cytotoxicity assay was used to detect the effects of different concentrations of glucocorticoid on the killing activity of B-cell tumor cell lines.

**Results:**

In this study, the CD19 CAR transfection rate of CD19 CAR-T cells was (51.34±5.28)％. The killing activities of different doses of methylprednisolone on Nalm6, Pfeiffer, and U2932 tumor cells were higher than that of dexamethasone at 24 h. The killing activities of 4 mg/mL methylprednisolone on Nalm6, Pfeiffer, and U2932 were higher than that of 0.75 mg/ml group, while the killing activity of 12 mg/ml methylprednisolone was lower than that of 2.25 mg/ml dexamethasone at 48 h. However, the killing activities of different doses of methylprednisolone on EHEB tumor cells were lower than those of different doses of dexamethasone at 24 and 48 h. The average MFI and proportion of CD19 CAR-T cells under different concentrations of glucocorticoid the proliferation inhibition of CD19 CAR-T cells by dexamethasone was higher than that of methylprednisolone. The proliferation inhibition of CD19 CAR-T cells of the two glucocorticoids in high concentration groups were more obvious than that in low concentration groups. When CD19 CAR-T cells were co-cultured with different tumor cells, the proportion and average MFI of CD19 CAR-T cells showed that the proliferation inhibition of dexamethasone was higher than that of methylprednisolone. The proliferation inhibition of CD19 CAR-T cells of the two glucocorticoids in high concentration groups was more obvious than that in low concentration groups.

**Conclusion:**

Dexamethasone inhibits the cell proliferation of CD19 CAR-T cells more than methylprednisolone during the targeting of different tumor cell lines. The inhibition effect of dexamethasone on the proliferation and amplification of CD19 CAR-T cells was greater than that of methylprednisolone during the targeting of CD19 CAR-T cells to different tumor cell lines. Moreover, the inhibition effect of the high dose group was more obvious.

嵌合抗原受体T细胞（CAR-T细胞）治疗在复发/难治血液系统和部分实体肿瘤的治疗上取得了重大突破[1]，患者可达到持久的完全缓解[2]。但是，CAR-T细胞治疗通常会出现细胞因子释放综合征（CRS）、CAR-T细胞相关脑病综合征、肿瘤溶解综合征等不良反应，严重时可危及生命[3]–[4]。针对CAR-T细胞治疗相关的毒副作用，糖皮质激素是重要的治疗选择之一[3]。但糖皮质激素的使用是否会影响CAR-T细胞的增殖，从而影响CAR-T细胞的临床疗效尚无研究确证。本研究我们对此进行了相关基础研究。

## 材料与方法

1. 细胞及主要试剂：急性B淋巴细胞白血病细胞株Nalm6、弥漫大B细胞淋巴瘤细胞株Pfeiffer及U2932、慢性B淋巴细胞白血病细胞株EHEB均购自美国模式培养物集存库（ATCC）；CD3磁珠分选试剂盒购自德国美天旎生物技术有限公司；CD19 CAR慢病毒及CD19 CAR流式检测抗体由上海吉倍公司提供；LDH毒性试剂盒、CFDA SE细胞增殖示踪荧光探针检测试剂盒均购自上海碧云天生物技术有限公司。

2. CD19 CAR-T细胞制备及慢病毒转染：采集6例健康志愿者外周血共30 ml，淋巴细胞分离液提取单个核细胞，CD3磁珠分选富集CD3^+^ T细胞。获得的细胞用含有IL-2、谷氨酰胺的T细胞专用培养基培养，培养第4天转染CD19 CAR慢病毒，上述细胞培养第12天收获CD19 CAR-T细胞，流式细胞术（FCM）检测CD19 CAR的转染效率。

3. 不同浓度的糖皮质激素对不同肿瘤细胞株体外杀伤活性的影响：以Nalm6、Pfeiffer、U2932、EHEB细胞为靶细胞（细胞密度均为1×10^6^/ml），加入不同浓度的糖皮质激素（0.75 mg/ml地塞米松、2.25 mg/ml地塞米松、4 mg/ml甲泼尼龙、12 mg/ml甲泼尼龙）后，置于含10％ FBS的RPMI 1640培养基，37 °C、饱和湿度、5％CO_2_培养箱中培养。按LDH细胞毒性检测试剂盒说明书进行操作，采用酶标仪检测490 nm处吸光度（*A*）值，计算其杀伤率。每组设3个复孔，实验重复3次。

4. 不同浓度的糖皮质激素对CD19 CAR-T细胞增殖的影响：CD19 CAR-T细胞与不同浓度的糖皮质激素（0.75 mg/ml地塞米松、2.25 mg/ml地塞米松、4 mg/ml甲泼尼龙、12 mg/ml甲泼尼龙）作用24、48 h分别采用FCM检测培养体系中CD19 CAR-T的比例；CFDA SE细胞增殖示踪荧光探针试剂染色后测定细胞的荧光强度，荧光强度增加表明对细胞增殖有抑制作用。实验复设3组，重复3次取均值。

5. CD19 CAR-T细胞杀伤不同肿瘤细胞株过程中不同浓度的糖皮质激素对其增殖的影响：将1×10^6^/ml的CD19 CAR-T细胞与Nalm6、Pfeiffer、U2932、EHEB细胞（细胞密度均为1×10^6^/ml）按1∶1混合培养。培养体系加入不同浓度的糖皮质激素（0.75 mg/ml地塞米松、2.25 mg/ml地塞米松、4 mg/ml甲泼尼龙、12 mg/ml甲泼尼龙）。在体系中加入CAR试剂、白细胞分化抗原CD19检测试剂及CD3检测试剂，FCM检测培养后的CD19 CAR-T细胞比例。CFDA SE细胞增殖示踪荧光探针试剂染色后测定荧光强度。实验复设3组，重复3次取均值。

6. 统计学处理：实验数据使用GraphPad Prism5.0和SPSS 17.0进行统计学处理。两组间比较采用独立样本*t*检验，多组间比较采用单因素方差分析，进一步两两比较采用LSD-*t*检验。*P*<0.05为差异有统计学意义。

## 结果

1. CD19 CAR-T细胞的转染效率：按CARTEST-19检测试剂盒说明书进行操作，流式细胞术检测6例健康志愿者T细胞来源CD19 CAR的转染率为（51.34±5.28）％。

2. 不同浓度的糖皮质激素对肿瘤细胞株的杀伤活性比较：不同浓度的糖皮质激素组间（0.75 mg/ml地塞米松和4 mg/ml甲泼尼龙组比较、2.25 mg/ml地塞米松和12 mg/ml甲泼尼龙组比较），培养24 h后，Nalm6、Pfeiffer、U2932细胞株中甲泼尼龙杀伤活性均明显高于地塞米松。培养48 h后，低剂量甲泼尼龙（4 mg/ml）对Nalm6、Pfeiffer、U2932细胞株的杀伤活性高于低浓度地塞米松（0.75 mg/ml），而高浓度甲泼尼龙（12 mg/ml）对Nalm6、Pfeiffer、U2932细胞株的杀伤活性低于高浓度地塞米松（2.25 mg/ml）。与Nalm6、Pfeiffer、U2932细胞株不同，在EHEB细胞株中共培养24、48 h后，两个浓度的甲泼尼龙组对肿瘤细胞株的杀伤活性均低于对应浓度的地塞米松组，差异有统计学意义（表1）。

**表1:** 不同浓度糖皮质激素对肿瘤细胞株的杀伤活性（％，*x*±*s*）

组别	Nalm6细胞	Pfeiffer细胞	U2932细胞	EHEB细胞
24h				
0.75mg/ml地塞米松	8.22±0.63	17.15±0.65	16.80±2.05	13.95±0.66
4mg/ml甲泼尼龙	41.49±1.80^a^	37.86±0.69^a^	35.51±1.00^a^	4.76±1.45^a^
2.25mg/ml地塞米松	4.43±1.54	5.77±1.08	5.10±1.01	10.12±2.19
12mg/ml甲泼尼龙	25.24±1.48^b^	23.17±0.82^b^	21.15±0.83^b^	2.18±2.71^b^
48h				
0.75mg/ml地塞米松	35.19±2.42	27.73±1.85	24.70±1.82	71.84±1.55
4mg/ml甲泼尼龙	69.68±3.43^a^	65.14±3.74^a^	57.48±4.60^a^	48.72±2.84^a^
2.25mg/ml地塞米松	21.43±2.19	13.92±2.62	13.22±1.12	92.63±1.32
12mg/ml甲泼尼龙	12.42±2.45^b^	3.24±2.39^b^	3.90±1.29^b^	30.43±1.58^b^

注：与0.75 mg/ml地塞米松比较，^a^*P*<0.05；与2.25 mg/ml地塞米松比较，^b^*P*<0.05。每组设3个复管，实验重复3次

3. 糖皮质激素作用下CD19 CAR-T细胞的扩增或增殖比较：CD19 CAR-T细胞在不同浓度的两种糖皮质激素（剂量同上）作用24、48 h后，共培养体系中CD19 CAR-T细胞比例与阴性对照组（CD19 CAR-T细胞）相比明显降低，提示在糖皮质激素作用下CD19 CAR-T细胞扩增被抑制。不同糖皮质激素组间比较，地塞米松较甲泼尼龙对CD19 CAR-T细胞扩增的抑制明显。0.75 mg/ml地塞米松组中的CD19 CAR-T细胞扩增比例低于4 mg/ml甲泼尼龙组，2.25 mg/ml地塞米松组低于12 mg/ml甲泼尼龙组。且无论地塞米松还是甲泼尼龙，高浓度组CD19 CAR-T细胞比例较低浓度组更低，提示高浓度糖皮质激素对CD19 CAR-T细胞扩增的抑制作用更明显（表2）。

共培养体系中CD19 CAR-T细胞平均荧光强度明显高于阴性对照组，提示糖皮质激素对CAR-T细胞增殖具有抑制作用。不同糖皮质激素对CD19 CAR-T细胞均有增殖抑制作用，0.75、2.25 mg/ml地塞米松组CD19 CAR-T细胞平均荧光强度分别高于4、12 mg/ml甲泼尼龙组，差异有统计学意义。且无论地塞米松还是甲泼尼龙，高浓度组CD19 CAR-T细胞平均荧光强度均高于低浓度组，提示高浓度糖皮质激素对CD19 CAR-T细胞的增殖抑制作用更明显，差异有统计学意义（表2）。

**表2:** 糖皮质激素对CD19 CAR-T细胞比例与荧光强度的影响（％，*x*±*s*）

组别	细胞比例	平均荧光强度
24h		
阴性对照	14.81±1.45	14568±18
0.75mg/ml地塞米松	9.40±0.82^a^	20892±21^a^
4mg/ml甲泼尼龙	11.97±0.47^ab^	18892±837^ab^
2.25mg/ml地塞米松	2.03±0.26^ab^	22508±68^ab^
12mg/ml甲泼尼龙	4.95±0.54^acd^	20841±534^acd^
48h		
阴性对照	19.56±0.61	13539±10
0.75mg/ml地塞米松	9.29±0.53^a^	20645±5^a^
4mg/ml甲泼尼龙	11.52±0.36^ab^	18978±470^ab^
2.25mg/ml地塞米松	1.61±0.31^ab^	23724±27^ab^
12mg/ml甲泼尼龙	3.57±0.45^acd^	22390±445^acd^

注：CAR-T细胞：嵌合抗原受体T细胞；与阴性对照组比较，^a^*P*<0.05；与0.75 mg/ml地塞米松比较，^b^*P*<0.05；与4 mg/ml甲泼尼龙比较，^c^*P*<0.05；与2.25 mg/ml地塞米松比较，^d^*P*<0.05。实验设3个复管，实验重复3次

4. 糖皮质激素作用下肿瘤细胞与CD19 CAR-T细胞共培养中CD19 CAR-T细胞的扩增或增殖情况：CD19 CAR-T细胞与Nalm6、Pfeiffer、U2932、EHEB细胞共培养体系中，加入不同浓度/种类糖皮质激素培养24、48 h。与阴性对照组相比（CD19 CAR-T细胞+肿瘤细胞），糖皮质激素共培养体系中CD19 CAR-T细胞比例均降低，提示CD19 CAR-T细胞与肿瘤细胞共培养体系中，不同浓度糖皮质激素抑制了CD19 CAR-T细胞的扩增。0.75 mg/ml地塞米松组中的CD19 CAR-T细胞比例低于4 mg/ml甲泼尼龙组、2.25 mg/ml地塞米松组CD19 CAR-T细胞比例低于12 mg/ml甲泼尼龙组，差异有统计学意义。不同糖皮质激素的组内比较，无论地塞米松或甲泼尼龙，高浓度组CD19 CAR-T细胞比例均低于低浓度组（表3）。

与阴性对照组相比，糖皮质激素共培养体系中CD19 CAR-T细胞平均荧光强度均增高，提示不同浓度糖皮质激素对CD19 CAR-T细胞的增殖具有抑制作用。0.75、2.25 mg/ml地塞米松组CD19 CAR-T细胞平均荧光强度分别高于4、12 mg/ml甲泼尼龙组。无论地塞米松或甲泼尼龙，高浓度组CD19 CAR-T细胞平均荧光强度均高于低浓度组（表3）。

**表3:** 糖皮质激素作用下肿瘤细胞与CD19 CAR-T细胞共培养体系中CD19 CAR-T细胞比例与荧光强度（％，*x*±*s*）

组别	CD19CAR-T细胞比例				CD19CAR-T细胞荧光强度			
	Nalm6细胞	Pfeiffer细胞	U2932细胞	EHEB细胞	Nalm6细胞	Pfeiffer细胞	U2932细胞	EHEB细胞
24h								
阴性对照	19.52±1.04	19.46±1.41	19.61±0.95	23.21±0.93	17948±10	16371±15	15565±471	15925±7
0.75mg/ml地塞米松	12.56±1.05^a^	13.27±1.14^a^	10.89±1.14^a^	13.63±0.56^a^	22289±14^a^	24056±41^a^	19605±12^a^	24793±149^a^
4mg/ml甲泼尼龙	16.36±0.71^ab^	16.28±0.76^ab^	14.62±0.85^ab^	16.90±0.71^ab^	20289±14^ab^	20506±470^ab^	17939±473^ab^	23346±469^ab^
2.25mg/ml地塞米松	3.50±0.58^ab^	7.54±0.73^ab^	6.62±1.23^ab^	9.89±1.61^ab^	27557±13^ab^	25587±18^ab^	25274±6^ab^	26554±73^ab^
12mg/ml甲泼尼龙	6.35±0.62^acd^	10.56±0.70^acd^	10.32±0.91^acd^	13.43±0.42^acd^	25546±828^acd^	23587±826^acd^	22607±477^acd^	21020±473^acd^
48h								
阴性对照	27.54±1.07	27.73±0.56	27.68±0.66	31.78±0.96	15756±5	14539±10	14448±11	14929±8
0.75mg/ml地塞米松	11.70±1.45^a^	10.05±2.14^a^	10.50±2.00^a^	12.10±1.29^a^	19925±9^a^	19054±5^a^	21733±6^a^	19111±9^a^
4mg/ml甲泼尼龙	16.00±0.75^ab^	15.06±0.87^ab^	15.12±1.33^ab^	15.57±1.31^ab^	18592±478^ab^	16721±470^ab^	20682±279^ab^	18111±9^ab^
2.25mg/ml地塞米松	2.68±0.51^ab^	3.81±1.27^ab^	2.41±0.57^ab^	8.40±0.58^ab^	20775±7^ab^	20119±8^ab^	23400±6^ab^	19924±9^ab^
12mg/ml甲泼尼龙	6.49±1.06^acd^	8.40±1.50^acd^	6.96±1.67^acd^	11.85±0.85^acd^	19768±15^acd^	18452±478^acd^	22400±6^acd^	18924±9^acd^

注：CAR-T细胞：嵌合抗原受体T细胞；与阴性对照组比较，^a^ *P*<0.05；与0.75 mg/ml地塞米松比较，^b^*P*<0.05；与4 mg/ml甲泼尼龙比较，^c^*P*<0.05；与2.25 mg/ml地塞米松比较，^d^*P*<0.05。实验设3个复管，实验重复3次

## 讨论

CD19 CAR-T细胞疗法迄今已经较为成熟，CAR-T细胞治疗后的毒副作用可从轻微的症状到伴有多器官功能衰竭。CRS轻者可能表现为轻度且呈自限性，包括发烧、肌肉痛、关节痛、食欲减退和疲劳；CRS严重者有可能危及生命，包括持续显著升高的体温、低血压、缺氧、凝血障碍以及脏器功能障碍[5]。IL-6受体抑制剂和糖皮质激素已经成为治疗高级别CRS的首选药物，可成功减轻严重CRS导致的副作用，并降低CAR-T细胞治疗副作用相关的死亡率[6]。免疫效应细胞相关神经毒性综合征（ICANS）最早表现是震颤、书写困难、表达能力轻度困难、定向障碍、注意力不集中、轻度嗜睡、头痛及表达性失语[7]–[8]，严重者发展为失语、肌阵挛、意识水平低下、癫痫、脑水肿[9]–[10]。大多数病例通过支持治疗和糖皮质激素的早期干预可缓解[11]。虽然IL-6受体抑制剂也可以在早期干预逆转ICANS，但IL-6受体抑制剂不能通过血脑屏障[12]，因此ICANS严重时糖皮质激素是首选治疗方法[13]。当患者存在神经毒性时，应接受地塞米松或甲泼尼龙治疗，直至症状改善[11],[14]。虽然严重不良反应相关症状得到控制，细胞因子水平迅速下降，临床症状迅速缓解，但同时也抑制了其CAR-T细胞的扩增和持久性，可能影响临床结局[15]。在应用糖皮质激素时，可能会抑制CAR-T细胞的体内扩增[16]。

最近一项针对复发/难治急性B淋巴细胞白血病CD19 CAR-T细胞治疗的临床研究显示，针对治疗副作用采用了糖皮质激素治疗，甚至是高浓度糖皮质激素，并不影响CAR-T细胞的疗效以及其增殖、体内持续时间[17]。而其结果与之前的文献报道不同，其原因可能在于该研究中均为短疗程使用高浓度糖皮质激素，平均应用4 d，91.3％患者应用≤7 d。但CAR-T细胞体内扩增和毒性的影响因素包括患者特异性因素和治疗相关因素[14]，例如疾病负担、化疗方案强度以及CAR-T细胞的输注剂量[18]。因此，糖皮质激素在CD19 CAR-T细胞治疗相关副作用中的应用中，特别是在不同B细胞恶性血液病的疗效，还需进一步探索。

在我们的基础实验中，24 h后不同剂量的甲泼尼龙对Nalm6、Pfeiffer及U2932细胞的杀伤活性高于地塞米松，48 h后低浓度甲泼尼龙对Nalm6、Pfeiffer、U2932杀伤活性高于低浓度地塞米松，而高浓度甲泼尼龙杀伤活性低于高浓度地塞米松；但24、48 h不同剂量的甲泼尼龙组对EHEB细胞的杀伤活性分别低于相应剂量的地塞米松。不同浓度的糖皮质激素对CD19 CAR-T细胞的扩增抑制作用，地塞米松组均高于甲泼尼龙组；两种糖皮质激素的高浓度组较低浓度对CD19 CAR-T细胞的扩增抑制更明显。CD19 CAR-T细胞在不同浓度的糖皮质激素作用下的平均荧光强度比较，地塞米松组低于甲泼尼龙组，即对CAR-T细胞的增殖抑制地塞米松高于甲泼尼龙；两种糖皮质激素的高浓度组较低浓度对CD19 CAR-T细胞的增殖抑制更明显。CD19 CAR-T细胞与不同肿瘤细胞共培养体系中，不同浓度的地塞米松组CD19 CAR-T细胞扩增比例低于甲泼尼龙组；两种糖皮质激素的高浓度组较低浓度CD19 CAR-T细胞的扩增比例为低。CD19 CAR-T细胞与不同肿瘤细胞共培养，其平均荧光强度比较，地塞米松组低于甲泼尼龙组，即对CAR-T细胞的增殖抑制地塞米松高于甲泼尼龙。

综上所述，CD19 CAR-T细胞靶向不同肿瘤细胞株过程中糖皮质激素对CD19 CART细胞的扩增有抑制作用。地塞米松对CD19 CAR-T细胞的扩增和增殖抑制作用大于甲泼尼龙，高浓度组该抑制作用更加明显。
